# A Study of the Immunoloregulation of Double Filtration Plasmapheresis in Maintenance Hemodialysis Patients with Chronic Hepatitis C

**DOI:** 10.1371/journal.pone.0082524

**Published:** 2013-12-16

**Authors:** Hongdi Cao, Ping Wen, Hong Ye, Zhiping Sun, Xia Shen, Xiaochun Wu, Chunsun Dai, Junwei Yang

**Affiliations:** Center for Kidney Disease, The Second Affiliated Hospital of Nanjing Medical University, Nanjing, Jiangsu Province, China; Saint Louis University, United States of America

## Abstract

Although a large number of drugs have been used to treat chronic hepatitis C (CHC), there still remains a great challenge to treat maintenance hemodialysis (MHD) patients with chronic hepatitis C. To clarify the immunnoloregulation of double filtration plasmapheresis (DFPP) in MHD patients with CHC, DFPP was performed in 20 MHD patients with CHC (HCV-antibody positive, serum HCV RNA >500 IU/ml more than 6 months and HCV genotype 1b). The clinical data was collected and peripheral blood mononuclear cells (PBMCs) were analyzed by flow cytometry at the time of hour 0, hour 3, day 3, day 7 and day 28 after the DFPP, respectively. Serum HCV particles could be removed partially by the DFPP. The titer of serum HCV RNA could remain in a lower level even 28 days after the treatment. Compared to MHD patients without HCV infection, the frequencies of innate immune cells were similar in MHD patients with CHC, while Th1/Th2 was elevated and the frequencies of regulatory T (Treg) cells were higher in those MHD patients with CHC. The frequencies of monocytes and natural killer (NK) cells remained after the DFPP in MHD patients with CHC. There were no significant changes of Th1, Th2 and Th1/Th2 in PBMC after DFPP. DFPP could reduce the frequencies of Th17 cells and Treg cells in PBMC from 7 days after DFPP in MHD patients with CHC. DFPP could partially remove the serum HCV particles mechanically. The titer of HCV RNA could remain in a lower level at least for 28 days probably due to the redistribution of the immunocytes in circulation.

## Introduction

Chronic hepatitis C (CHC) is the main cause of chronic liver disease in maintenance hemodialysis (MHD) patients who are at particular high risk for hepatitis C virus (HCV) infection. Among MHD patients, the prevalence of CHC varies greatly, from less than 5% to nearly 60% according to different areas of the world [1]–[5]. The prevalence of HCV infection has declined in many dialysis centers, and yet it remains unacceptably high, ranging from 8% to 10% even in the industrialized countries [6]. It was recommended to monitor the markers of HCV routinely in MHD patients [7]. What’s more, it has also been reported that HCV was associated with higher all-cause and cardiovascular mortality in MHD patients [8]. Over the past decades, several studies have pointed that the effective strategies of preventing and treating HCV infection in MHD patients could improve the prognosis of this population [8].

Combination of ribavirin (RBV) with peginterferon (PEG-IFN) is considered the gold standard of therapy in HCV-positive patients with normal renal function based on sustained virus response (SVR) up to 50% to 60% [9].The distribution of HCV genotypes were geographical different, and the predominant HCV genotype in China was genotype 1, with type 1b in particular [10], which was similar in MHD patients [11]. Unfortunately, SVR to standard therapy was much lower in patients with HCV genotype 1. Physicians are reluctant to use RBV in MHD patients given the fear of the drug-related side effects, particularly hemolytic anemia, which can be exacerbated in MHD patients [12]. The risk of severe side effects and the SVR limited the application of RBV and PEG-IFN in MHD patients. To date, it has been still difficult to treat CHC in MHD patients.

HCV clearance is mediated by T cells and the innate immune response. However, due to the progressive loss of kidney function, the function and interactions of the innate and adaptive immune systems in MHD patients are impaired and become much more complex[13], [14]. Thus, it seems that improving the impairment of the innate and adaptive immune systems might provide novel treatment strategy for MHD patients with CHC. DFPP, a newly developed apheretic technique, selectively remove high molecular weight substances, has been proven to have several beneficial effects in immune systems. Recently, it has been reported that double-filtration plasmapheresis (DFPP) was effective for CHC. For CHC patients with high viral load, DFPP and IFN combination therapy produced a great reduction of viral load during the early stage of treatment and achieved a high SVR [15]. However, as it stands, DFPP has also not been used in MHD patients with CHC and the underlying mechanisms of DFPP remain largely unknown.

In this study, single DFPP without IFN or RBV was given to MHD patients with CHC and the immune regulation of DFPP was focused. To clarify the immune regulation of DFPP in MHD patients with CHC, innate and adaptive immune cells in peripheral blood mononuclear cells (PBMCs) were monitored during the DFPP. It might provide the immunological mechanisms of a useful adjuvant therapy in MHD patients with CHC.

## Materials and Methods

### Ethics statement

All of the following details of the study were approval by the responsible ethics committee of Nanjing Medical University (Permit Number: KY027). The written informed consent was supplied by the patients before the study.

### Study population

From October 2011 to April 2012, twenty MHD patients with CHC and 8 MHD patients without CHC from the Center for Kidney Disease of 2^nd^ Affiliated Hospital of Nanjing Medical University were recruited. MHD patients with CHC were defined as MHD patients with HCV-antibody positive and the titer of HCV RNA more than 500 IU/ml for 6 months or longer. The HCV genotype was genotype 1b in these 20 MHD patients with CHC. MHD patients without CHC were defined as MHD patients with HCV-antibody negative and the titer of HCV RNA less than 500 IU/ml. All participants were negative for hepatitis B surface antigen. Patients with platelet counts less than 100*10^9^/L and leukocyte counts less than 3*10^9^/L were excluded from the study.

### DFPP

DFPP was performed once in MHD patients with CHC. For the DFPP, a Plasmaflo™ OP-08W (Asahi Kasei Medical, Tokyo, Japan) was used to separate the blood into plasma and cell components. The virus was then removed from the separated plasma by a second filter, Cascadeflo™ EC-50W (Asahi Kasei Medical, Tokyo, Japan) with an average pore size of 30 nm. The final volume of treated plasma was 50 ml/kg, and the treatment time was about 3 hours for the DFPP. All the patients were given the single DFPP. Blood samples were obtained at the time of hour 0, hour 3, day 3, day 7 and day 28 of the DFPP.

### HCV RNA measurement

The titer of serum HCV RNA was measured by real-time PCR with diagnostic kit for quantification of HCV RNA (PCR-Fluorescence Probing, Shanghai, China), which has a lower limit of detection of 500 IU/ml. Serum HCV RNA levels were measured by real-time PCR before the DFPP (hour 0), after the DFPP(hour 3) and 28 days after the therapy. The quantity of HCV RNA was converted to a log value at each virus measurement point.

### Isolation of PBMCs and flow cytometry

PBMCs were isolated from fresh heparin anti-coagulated blood by density gradient centrifugation using Ficoll-Hypaque (Sigma Chemical Co., St Louis, MO, USA). Multi-parameter flow cytometry was performed using a BD FACSAira instrument for detection of single fluorochromes and analyzed using FACSDiva software (BD Biosciences, San Jose, CA, USA).

Fluorochrome-labeled monoclonal antibodies (MAbs) specific for CD14-FITC, CD3-FITC CD16CD56-PE, CD3-PerCP-Cy5-5, CD8-APC, IFNγ-FITC, IL-4-PE, CD4-PerCP-Cy5-5, CD25-FITC, CD127-PE were supplied by Beckman Coulter (San Diego, CA, USA). IL-17-PE MAb was purchased from eBiosciences (San Diego, CA, USA). For the detection of IL-17, IFNγ and IL-4, PBMC (100 µl) was firstly activated with 50 ng/ml phorbol-12-myristate 13-acetate (PMA),1 µg/ml ionomycin and 1.7 µg/ml monensin ( all from Sigma Chemical Co., St Louis, MO, USA) for 4 hours. After staining for surface antigens, the remaining cells were permeabilized and stained with IFNγ-FITC, IL-4-PE or IL-17-PE, respectively. The cells were fixed in 1% of paraformaldehyde if they could not be detected immediately.

The frequency of monocytes was expressed as a ratio of CD14+ cells to PBMCs, while natural killer (NK) cells was expresses as a ratio of CD3-(CD16CD56)+ cells to PBMCs. For subsets of T helper (Th) lymphocytes, the frequencies of Th1 and Th2 cells were expressed as a ratio of IFN+CD4+ cells to CD4+ lymphocytes and IL-4+CD4+ cells to CD4+ lymphocytes, respectively. The frequency of Th17 cells was expressed as a ratio of CD4+IL-17A+ cells to CD4+ lymphocytes. The frequency of regulatory T (Treg) lymphocytes was expressed as a ratio of CD4+CD25+CD127^−/low^ cells to CD4+ lymphocytes.

### Statistic analysis

The data was analyzed using SPSS for windows version 18.0. Clinical data are expressed as the mean ± SD or frequency, as appropriate. Comparisons of continuous data of patients used the independent *t* test. Compared *t* test was used in the data at different time point after the DFPP to the data before the DFPP. A *P* value less than 0.05 was considered to represent a statistically significant difference.

## Results

### The clinical characteristics of MHD patients with CHC and without CHC

This study included 20 MHD patients with CHC and 8 MHD patients without CHC. Baseline characteristics of participants were summarized in **Table 1** **.** The age of MHD patients with CHC was 50.7±2.3 years old and male-to-female ratio was 12:8; while the age of MHD patients without CHC was 41.7±10.9 years old and male-to-female ratio was 5:3. The duration of hemodialysis in MHD patients with CHC was longer than those without CHC, with 11.0±6.5 years compared to 6.4±5.4 years (P<0.05). Hemoglobin (HGB) was similar in these two groups, with 110.5±7.9 g/L in MHD patients without CHC and 108.2±15.2 g/L in patients with CHC. Liver function in MHD patients without CHC was all within the normal range, while 4 patients with CHC had mild abnormal liver function. Serum alanine transaminase (ALT) was 37.3±28.0 U/L in patients with CHC compared to 20.3±7.8 U/L in patients without CHC. There was no difference in serum albumin (ALB) between MHD patients with and without CHC. The titer of serum HCV RNA in MHD patients with CHC before DFPP was 0.53*10^6^ IU/ml –50.4*10^6^ IU/ml.

**Table 1:** The characteristics of MHD patients without and with CHC.

Clinical characteristics	MHD patients without CHC	MHD patients with CHC
No. of Cases	8	20
Age(yr)	41.7±10.9	50.7±2.3
Gender ratio(M:F)	5:3	12:8
HD vintage (yrs)*	6.4±5.4	11.0±6.5
Hemoglobin (g/L)	110.5±7.9	108.2±15.2
ALT (U/L)	20.3±7.8	37.3±28.0
ALB (g/L)	42.4±1.98	43.1±1.97
HCV RNA (*10^ 6 ^IU/ml)	undetectable	0.53–50.4

P<0.05.

### The safety of DFPP

When DFPP was performed in those 20 MHD patients with CHC, 4 of 20 (20.0%) cases experienced some adverse events. A drop in blood pressure was observed in 2 patients, but recovered after giving intravenous 100 ml–200 ml saline solution. Minor disorder was observed in 2 patients, which was temporary and recovered without any treatment. All the 20 patients completed the treatment smoothly. Clinical markers were monitored during the DFPP. There was no change in HGB and the platelet count. The fibrinogen levels fell significantly from 2.68±0.76 g/L to 1.34±0.7 g/L after DFPP. Serum ALB was reduced from 42.9±2.1 g/L to 34.5±3.1 g/L after DFPP. Serum immunoglobulin could also be removed by DFPP.The reduction of fibrinogen, ALB and immunoglobulin could recover to the initial levels within one week after the completion of DFPP. There was no bleeding and infection or other adverse events within one month after the DFPP.

### Single DFPP could maintain the titer of serum HCV RNA in a lower level for at least 28 days

**Figure 1:**
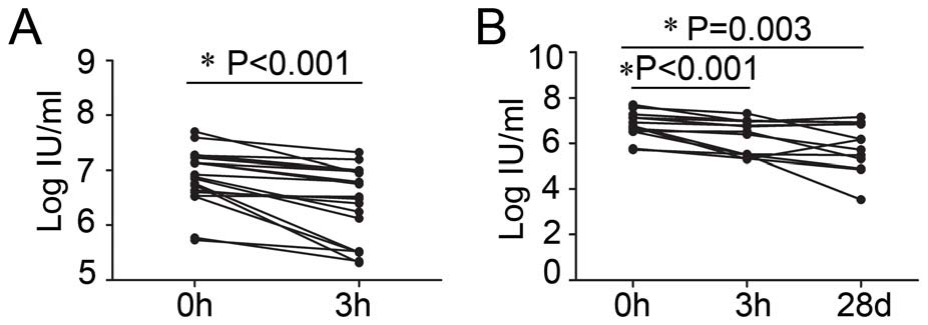
Viral dynamics of HCV RNA during the DFPP in MHD patients with CHC. A. The titers of HCV RNA before and after the DFPP. B. The titers of HCV RNA during 28 days after DFPP. The Y axis indicates the log HCV RNA. The X axis indicates the time course of the therapy. 0 h means before the DFPP, 3 h means after the DFPP, 28 d means 28 days after the DFPP.

Serum HCV RNA was used to evaluate the efficiency of DFPP. The titer of serum HCV RNA was monitored before and after the DFPP. The titer of serum HCV RNA could be reduced from 0.53*10^6^ IU/ml –50.4*10^6^ IU/ml to 0.20 *10^6^ IU/ml –21*10^6^ IU/ml in MHD patients with CHC by single DFPP. The titer of serum HCV RNA reduced rapidly after the therapy, which was accordant to the reported. [15] The log HCV RNA before the DFPP was 6.88±0.51 IU/ml, while which was lower after the DFPP with log HCV RNA 6.43±0.67 IU/ml (P<0.001) (Figure 1A). There was another important phenomenon we were interested. The level of serum HCV RNA was monitored 28 days after the DFPP. The log HCV RNA on day 28 after DFPP was 5.92±1.07 IU/ml, which was still lower than the titer before DFPP (P = 0.003) (Figure 1B). It was demonstrated that the titer of serum HCV RNA was maintained in a lower level even 28 days after the therapy.

### The frequencies of monocyte in PBMC in MHD patients with CHC maintained during one single DFPP

**Figure 2:**
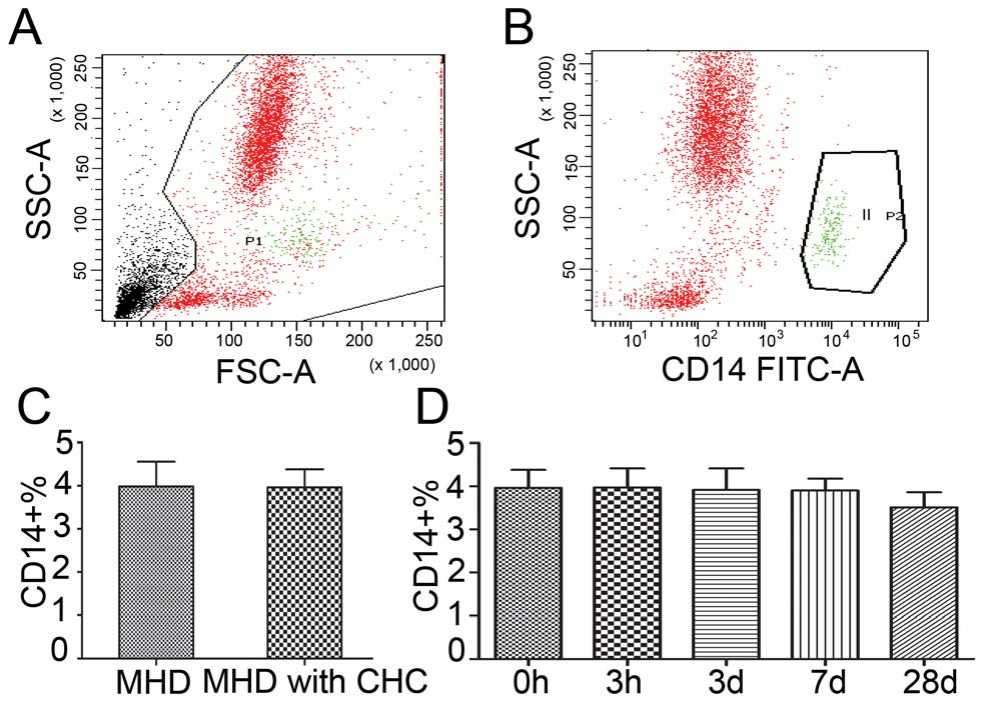
The frequencies of monocytes in PBMC after a single DFPP. A, B. Monocyte was expressed as CD14+ cells in PBMCs. C. The frequencies of monocytes in MHD patients with CHC compared to those without CHC. D. The frequencies of monocytes in MHD patients with CHC during the DFPP.

Monocyte is one of the important parts of the innate immune system in human, which play multiple roles in immune function. The classical monocyte is characterized by high level expression of the CD14 cell surface receptor. The frequency of monocyte was expressed as a ratio of CD14+ cells to PBMC (Figure 2 A, B). The frequency of monocyte in PBMC was evaluated in MHD patients with or without CHC. It was found that the frequency of monocytes was 3.99±1.52% in patients without CHC and 3.96±1.81% in patients with CHC (P = 0.983) (Figure 2 C). The tendency of monocyte in PBMC was monitored in MHD patients with CHC during the DFPP. It was shown that the frequencies of monocytes in PBMC maintained during the DFPP, with 3.97±1.88% at hour 3, 3.92±1.80% on day 3, 3.90±1.20% on day 7 and 3.51±1.52% on day 28, respectively (P>0.05) (Figure 2 D).

### The frequencies of NK cells remained unchanged during single DFPP

**Figure 3:**
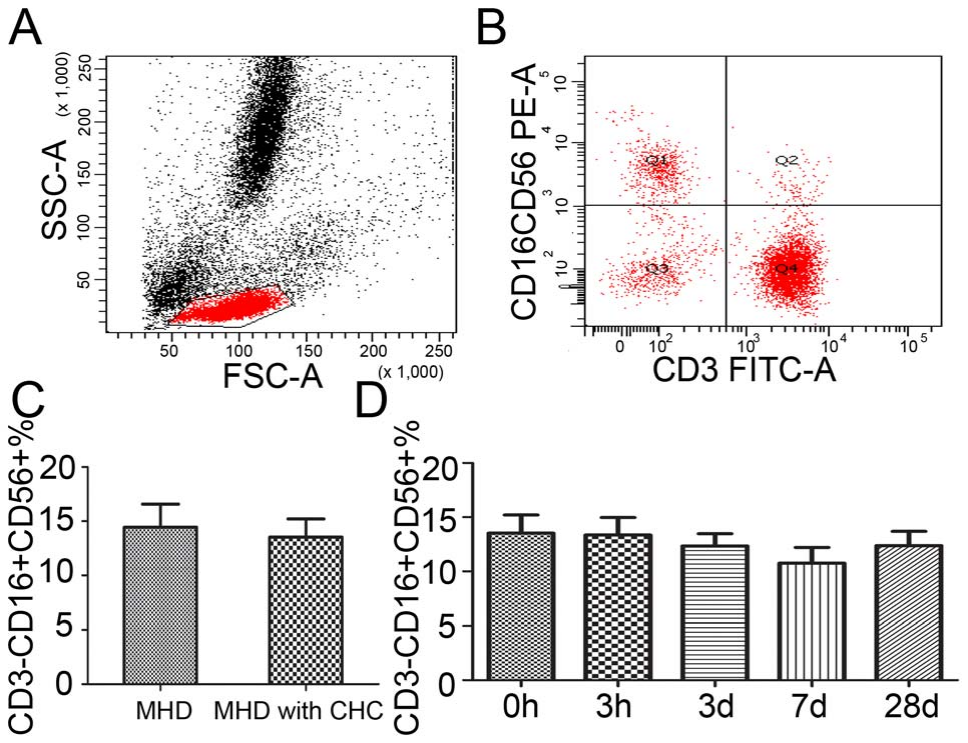
The percentages of NK cells in PBMC after the DFPP. A, B. NK cell was expressed as CD3–CD16+CD56+ cells in PBMCs. C. The frequencies of NK cells in MHD patients with CHC compared to those without CHC. D. The frequencies of NK cells in MHD patients with CHC during the DFPP.

NK cells are a type of cytotoxic lymphocytes critical to the innate immune system. The frequency of NK cells was expressed as a ratio of CD3–CD16+CD56+ cells to PBMC (Figure 3 A, B). The frequency of NK cells was 14.4±5.3% in patients without CHC and 13.5±7.3% in those with CHC (P = 0.346) (Figure 3 C). There were no significant changes of NK cells during the DFPP in MHD patients with CHC, with 13.4±7.2% at hour 3, 12.3±5.1% on day 3, 10.8±6.4% on day 7 and 12.4±5.7% on day 28, respectively (P>0.05 ) (Figure 3 D).

### There were no significant changes of Th1, Th2 and Th1/Th2 in PBMC during the DFPP

**Figure 4:**
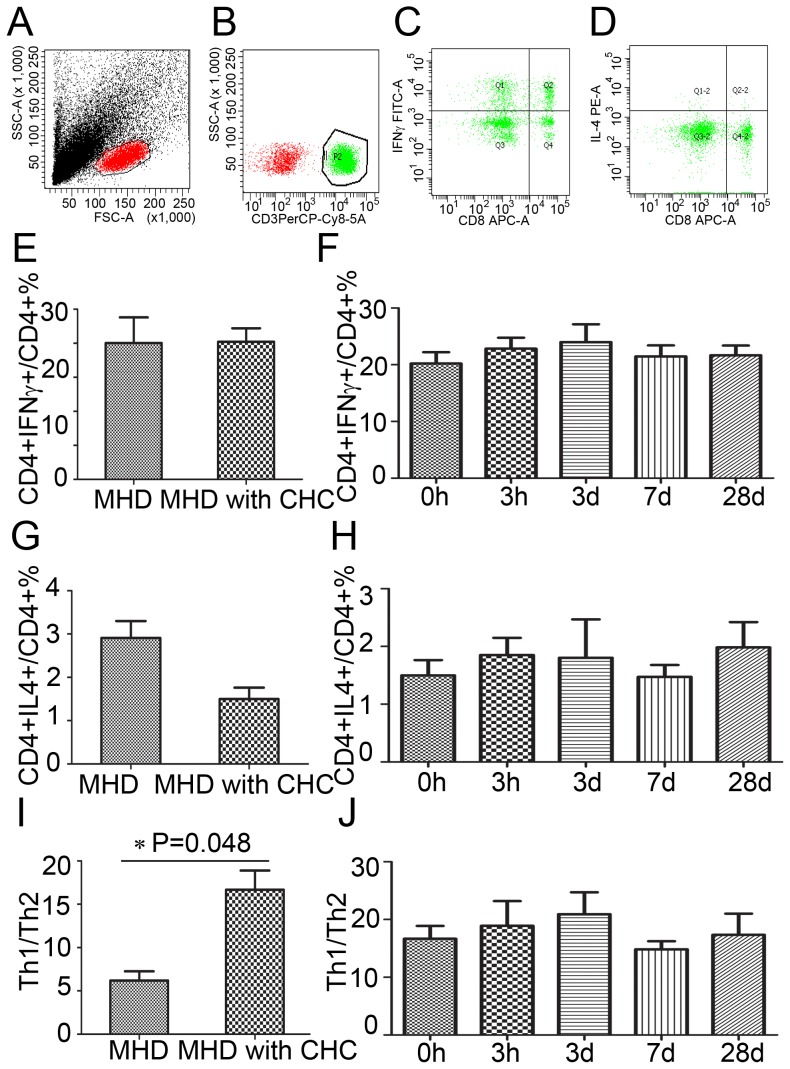
The tendencies of Th1 and Th2 cells in PBMC after the DFPP. A-D. Th1 cells was expressed as IFN-γ+CD4+ cells; Th2 cells was expressed as IL-4+CD4+ cells. E, F The frequencies of Th1 cells in MHD patients with CHC during the DFPP. G, H The frequencies of Th2 cells in MHD patients with CHC during the DFPP. I, J The ratio of Th1 to Th2 cells in MHD patients with CHC.

The frequencies of Th1 and Th2 cells were expressed as a ratio of IFN-γ+CD4+ cells to CD4+ lymphocytes and a ratio of IL-4+CD4+ cells to CD4+ lymphocytes, respectively (Figure 4 A-D). There was no significant difference of the frequencies of Th1 and Th2 cells in MHD patients with or without CHC (Figure 4 E, G). Th1/Th2 was enhanced in MHD patients with CHC compared to those MHD patients without CHC, 16.7±8.9 *vs* 6.2±2.6 (P = 0.048) (Figure 4 I). The frequencies of Th1 cells, the frequencies of Th2 cells and the ratio of Th1 to Th2 in MHD patients with CHC remained unchanged during the DFPP, respectively (Figure 4 F, H, J). It was demonstrated that there was no significant change of Th1 cells and Th2 cells during the DFPP, although Th1/Th2 was enhance in MHD patients with CHC.

### The frequency of Th17 cells decreased from day 7 after the DFPP

**Figure 5:**
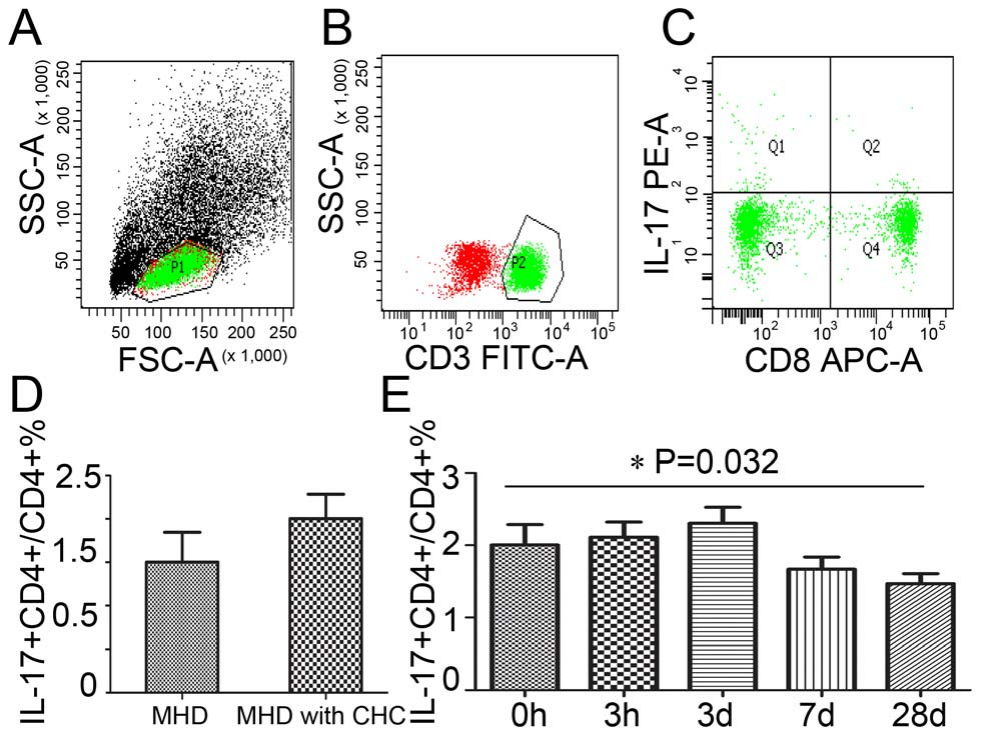
The changes of Th17 cells in PBMC after the DFPP. A-C. Th17 cell was expressed as CD17+CD4+ cells in PBMC. D. The frequency of Th17 cells in MHD patients with CHC compared to those without CHC. E. The frequencies of Th17 cells in MHD patients with CHC during the DFPP.

Th17 cells have been identified as a unique subset of T helper cells, which are CD4+ T cells that are defined by the production of IL17. The frequency of Th17 cells in PBMC was evaluated in patients during the DFPP, which was expressed as a ratio of IL-17+CD4+ cells to CD4+ lymphocytes (Figure 5 A-C). The frequency of Th17 cells was 2.00±1.19% in MHD patients with CHC and 1.50±0.91% in those patients without CHC, although there was no significant difference (P = 0.428) (Figure 5 D). The tendency of Th17 cells in PBMC was monitored during the DFPP. It was found that accompanied with the reduction of the titer of serum HCV RNA, the frequency of Th17 cells decreased from day 7 (1.67±0.73%) to day 28 (1.47±0.59%) after the therapy (P = 0.032) (Figure 5 E).

### The frequency of Treg cells was higher in MHD patients with CHC, which was reduced from 7 days after the DFPP

**Figure 6:**
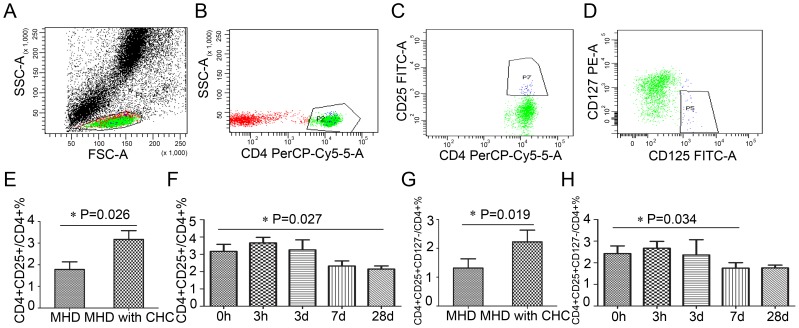
The tendencies of Treg cells in PBMC after a single DFPP. A-D. Treg cell was expressed as CD4+CD25+ CD127^low/−^cells in PBMC. E, F. The frequency of CD4+CD25+ Treg cells in MHD patients with CHC during the DFPP. G, H. The frequencies of CD4+CD25+ CD127^low/−^ Treg cells in MHD patients with CHC during the DFPP

Treg cells are a component of the immune system that suppress immune responses of other cells, which come in many forms with the most well-understood being those that express CD4, CD25, and Foxp3 (CD4+CD25+Foxp3+ regulatory T cells). It has recently been suggested that a lack of CD127 expression can be used to identify human CD4+ regulatory T cells, especially when combined with CD25. It has been reported that CD4+CD25+CD127^−/low^ phenotype is a good and easy-to-perform surrogate identification strategy for FOXP3+ regulatory T cells in HIV-1 infected subjects [16]. The frequency of Treg cells in this study was expressed as a ratio of CD4+CD25+CD127^−/low^ cells to CD4+ lymphocytes. The ratio of CD4+CD25+ cells to CD4+ lymphocytes was also analyzed (Figure 6 A-D).

The frequency of CD4+CD25+ cells in MHD patients with CHC was higher than that in MHD patients without CHC, 3.17±1.68% *vs* 1.78±0.86% (P = 0.026)(Figure 6 E). The frequency of CD4+CD25+CD127^−/low^ Treg cells was also higher in patients with CHC than in those without CHC, 2.22±1.47% *vs* 1.32±0.78% (P = 0.019) (Figure 6 G). The tendency of Treg cells was monitored during the DFPP. Accompanied with the reduction of serum HCV particles, the frequency of Treg cells decreased from day 7 to day 28 after the DFPP. The frequency of CD4+CD25+ Treg cells was 2.32±1.07% on day 7 and 2.15±0.78% on day 28 (P = 0.027) after the DFPP (Figure 6 F), while the frequency of CD4+CD25+CD127^−/low^ Treg cells was lower on day 7 with 1.75±0.95% after the DFPP (P = 0.034) (Figure 6 H).

## Discussion

More than 170 million people worldwide are chronically infected with HCV [17]. MHD patients are at particular high risk for bloodborne infections because of prolonged vascular access and potential for exposure to contaminated equipment. The prevalence of HCV in MHD patients exceeds 50% in some developing countries [18], [19]. Although it has been documented that CHC among MHD patients was mild in disease activity and was not so progressive compared to the controls with CHC[20], in MHD patients, HCV infection exhibited distinct clinical patterns, including associations with higher dialysis treatment vintage, and is associated with higher mortality[8].

Combination of RBV with PEG-IFN is considered the gold standard of therapy in HCV-positive patients with normal renal function [9]. Although this therapeutic schedule was also recommended in MHD patients with HCV, the risk of severe life-threatening side effects in this population could not be ignored [12] [21]. The high cost of the antiviral treatment, the risk of severe side effects, and the lack of data on the impact of therapy and SVR response on mortality in MHD patients limited the application in this population [22]. There is still a relative lack of treatment of CHC in MHD patients which needs further research. Finding better therapeutic measures to MHD patients with CHC has been a great challenge in improving the long-term prognosis of dialysis patients.

Immunological cell dysfunction is an important clinical feature in MHD patients, which results in an increased susceptibility to bacterial and viral infection. It has been reported that the absolute number of NK cells in MHD patients is markedly decreased (by 20–32%) [23]. Monocytes in MHD patients have an activated profile with increased secretion of proinflammatory cytokines[24]. The injury of T cell-mediated immune function is an important characteristic in MHD patients[25]. The effector T cells decreased severely and the inhibitory Treg cells reduced in MHD patients [14], [26]. In patients with CHC, HCV has several means of inhibiting innate immune mechanisms, including inhibiting type I interferon responses, raising the activation threshold of NK cells activation [27], [28]. It has been reported that the pro-inflammatory cells Th17 cells were increased with severity of liver inflammation in patients with CHC [29]. Higher frequency of Tregs was identified in patients with CHC compared with controls, which might inhibit Th1 and Th2 cell responses either indirectly by modulating antigen-presenting cell function or directly by cell–cell contact[30], [31]. The disturbance of immune function is more noticeable in MHD patients with CHC. Data in this study also demonstrated that, the frequency of Treg cells was higher and Th1/Th2 was increased in MHD patients with CHC compared to those MHD patients without CHC.

DFPP, a newly developed apheretic technique, selectively remove high molecular weight substances, including immunoglobulins and immune complexes, has been widely used to eliminate auto-antibodies from plasma. Recently, DFPP combined with IFN and RBV has been used in CHC patients with high viral load and living donor liver transplant recipients with hepatitis C. All these clinical studies demonstrated that DFPP appeared to be effective in reducing viremia and preventing HCV recurrence in patients. DFPP combined with IFN and RBV is indicated more for relapse patients than for NVR patients [32]–[34]. Kondo etc observed 12 CHC patients treated with DFPP/PEG-IFN/RBV therapy. The rapid reduction of HCV-Core antigens and changes in the distribution of lymphoid cells could contribute to the favorable immunological response during the DFPP/Peg-IFN/RBV therapy [35], [36]. However, the effect of DFPP in MHD patients with CHC has not been reported. Thus far, the immunological mechanism underlying the efficacy of DFPP besides the reduction of HCV RNA remains poorly understood. The understanding of its underlying mechanisms may identify novel avenues for treatment MHD patients with CHC.

In order to exclude the interference of IFN and RBV, single DFPP without antivirus drugs was performed in MHD patients with CHC. It was found that besides the rapid reduction of HCV particles, the titer of serum HCV RNA maintained in a lower level even 28 days after the therapy. Main immunocytes in PBMCs in MHD patients with CHC were monitored during the DFPP. Single DFPP could not affect the frequencies of innate immune cells such as monocytes and NK cells. There were no significant changes in the frequencies of Th1 cells, Th2 cells and Th1/Th2 during the therapy. The frequency of Th17 cells decreased from day 7 to day 28 after the DFPP. It was indicated that the systemic inflammatory reaction was ameliorated after the partial removal of HCV particles. An increased frequency of Treg cells at the onset of HCV infection was suggested to predict a chronic outcome of the infection [37]. In this study, we found that the frequencies of Treg cells decreased from day 7 after the DFPP. The decrease of Treg cells after the DFPP indicated that the immune tolerance was partially released accompanied with the reduction of HCV particles. To some extent, the decrease of Treg cells might contribute to the persistent lower titer of serum HCV RNA.

In conclusion, it was a preliminary study focusing on the improvement of immunological function in PBMCs after the DFPP in MHD patients with CHC. The application of DFPP with or without anti-virus drugs in MHD patients with CHC still needs further studied.
